# Rebleeding after placing over‐the‐scope clips: Rare complication of prophylactic closure after gastric endoscopic submucosal dissection

**DOI:** 10.1002/jgh3.12354

**Published:** 2020-05-13

**Authors:** Shintaro Fujihara, Hideki Kobara, Noriko Nishiyama, Hirohito Mori, Tsutomu Masaki

**Affiliations:** ^1^ Department of Gastroenterology and Neurology, Faculty of Medicine Kagawa University Kita‐gun Japan

## Abstract

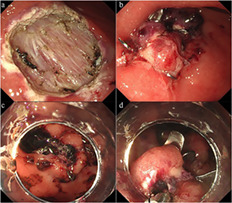

Monopolar hemostatic forceps may still be able to treat for rebleeding with above mentioned challenging characteristics, and it is earning a lace in the new enodscopic hemostasis repertoire.

A 79‐year‐old man underwent gastric endoscopic submucosal dissection (ESD) of a 20‐mm flat elevated adenoma in the lower gastric body (Fig. 1a). He had a history of coronary artery disease and chronic kidney disease and was receiving dual antiplatelet therapy, which was discontinued 1 week before ESD. He was considered to be at a high risk of delayed bleeding, as well as perforation, because of the thin muscle layer in the greater curvature of the gastric body. Therefore, we attempted prophylactic closure of the artificial ulcer using two over‐the‐scope clips (OTSCs) (Ovesco Endoscopy, Tubingen, Germany) (Fig. 1b). No procedure‐related complications occurred. Ten days after gastric ESD, the patient presented with fresh hematemesis and shock. Emergent endoscopy revealed Forrest Ib bleeding with clots near the OTSCs (Fig. 1c) and ruptured vessels and a small exposed defect near the OTSCs. Endoscopic hemostasis was performed with a monopolar hemostatic forceps (MHF) (Fig. 1d). The patient was treated with proton pump inhibitors, and anticoagulation therapy was discontinued postprocedure. Follow‐up esophagoduodenoscopy on day 5 showed no evidence of recurrent bleeding. The patient's symptoms resolved, and he was discharged in good clinical condition 10 days after endoscopic hemostasis.

**Figure 1:**
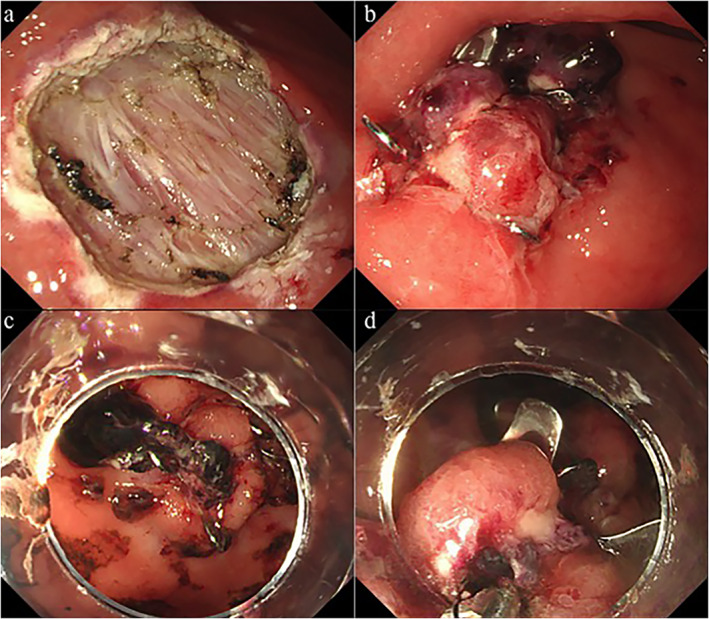
(a) A large mucosal defect after gastric endoscopic submucosal dissection. (b) Successful application of over‐the‐scope clips. (c) Forest 1b bleeding from the center of an over‐the‐scope clip placement area seen during water jet irrigation. (d) The affected area after coagulating the exposed vessels using a monopolar hemostatic forceps in the soft coagulation mode.

Multiple antithrombotic drug use, anticoagulant use, and heparin bridging therapy are high‐risk factors for delayed bleeding after gastric ESD.^1^ An endoscopic approach for prevention of delayed bleeding after ESD is described as follows: polyglycolic acid sheet and fibrin glue, prophylactic closure using endoclip and loop, OTSC, overstitch suturing, or hand‐sewn suturing.^1^ The OTSC has been mainly used to manage refractory bleeding, perforation, and fistula and was also applied to the closure of large mucosal defects after gastric ESD.^2^ While the use of OTSCs in gastrointestinal bleeding results in a high rate of primary hemostasis, rebleeding occurs postprocedure in 14.9% of patients receiving antithrombotic/anticoagulant therapy.^3^ However, the ideal secondary hemostatic method following OTSC placement is unknown. Standard endoscopic therapies for rebleeding are hemoclips, argon plasma coagulation, repeat OTSC placement, and epinephrine injections.^4^ The MHF is an effective device recently developed for complete hemostasis during ESD. Standard methods of hemostasis are often limited at bleeding sites with indurated tissues after OTSC placement. Consequently, using the MHF can be a favorable option. In conclusion, this case suggests that OTSC is not perfect for managing patients receiving antithrombotic/anticoagulant therapy and that MHF is a possible new endoscopic hemostatic approach for rebleeding after OTSC placement.
